# Population Analysis of the *Fusarium graminearum* Species Complex from Wheat in China Show a Shift to More Aggressive Isolates

**DOI:** 10.1371/journal.pone.0031722

**Published:** 2012-02-20

**Authors:** Hao Zhang, Theo Van der Lee, Cees Waalwijk, Wanquan Chen, Jin Xu, Jingsheng Xu, Ye Zhang, Jie Feng

**Affiliations:** 1 State Key Laboratory for Biology of Plant Diseases and Insect Pests, Institute of Plant Protection, Chinese Academy of Agriculture Sciences, Beijing, China; 2 Plant Research International, Wageningen, The Netherlands; Hungarian Academy of Sciences, Hungary

## Abstract

A large number of *Fusarium* isolates was collected from blighted wheat spikes originating from 175 sampling sites, covering 15 provinces in China. Species and trichothecene chemotype determination by multilocus genotyping (MLGT) indicated that *F. graminearum* s. str. with the 15-acetyl deoxynivalenol (15ADON) chemotype and *F. asiaticum* with either the nivalenol (NIV) or the 3-acetyl deoxynivalenol (3ADON) chemotype were the dominant causal agents. Bayesian model-based clustering with allele data obtained with 12 variable number of tandem repeats (VNTR) markers, detected three genetic clusters that also show distinct chemotypes. High levels of population genetic differentiation and low levels of effective number of migrants were observed between these three clusters. Additional genotypic analyses revealed that *F. graminearum* s. str. and *F. asiaticum* are sympatric. In addition, composition analysis of these clusters indicated a biased gene flow from 3ADON to NIV producers in *F. asiaticum*. In phenotypic analyses, *F. asiaticum* that produce 3ADON revealed significant advantages over *F. asiaticum* that produce NIV in pathogenicity, growth rate, fecundity, conidial length, trichothecene accumulation and resistance to benzimidazole. These results suggest that natural selection drives the spread of a more vigorous, more toxigenic pathogen population which also shows higher levels of fungicide resistance.

## Introduction

Fusarium head blight (FHB) or head scab is a devastating disease of wheat (*Triticum aestivum* L) and barley (*Hordeum vulgare* L) in China [1], [2], [3], [4] and many other regions of the world [5], [6]. FHB not only causes quantitative yield loss but may also reduce grain quality due to contamination of mycotoxins produced by the *Fusarium* pathogens [7]. Mycotoxin contamination poses a significant risk to food safety and animal health. A particular class of mycotoxins, the trichothecenes have been shown to inhibit eukaryotic protein synthesis and compromise the function of the immune system [8], [9].

In China, wheat is an important crop, second in importance only to rice [10]. FHB has been a problem in some provinces and in some years since 1936, when the first serious outbreak occurred [10]. During the past ten years, epidemic outbreaks have become more frequent and severe in China. The recent epidemics in 2003, 2008 and 2010 made wheat a less attractive crop for growers, resulting in a sharp decrease of wheat growing acreage. The disease frequently occurs in wheat growing areas in the middle and lower valleys of the Yangtze River and the mountainous areas in southwest China [3]. FHB has also become an increasing problem in the northern regions, and is now a threat in more than ten provinces including the most important agricultural regions of China. In recent years, FHB occurred on a large scale in several Northern provinces, such as Hebei, Shanxi and Shandong, where occurrence of this disease was rare in the past [10].

Many *Fusarium* species and *Microdochium nivale* are associated with FHB [11], [12], [13], [14]. *F. graminearum* sensu lato is the most frequently isolated causal agent of FHB in China. Phylogenetic species recognition, with genealogical concordance [15], has provided strong molecular genetic evidence that *F. graminearum* sensu lato comprises at least 15 biogeographically structured and phylogenetically distinct species, also known as the *Fusarium graminearum* species complex, FGSC. [16], [17], [18], [19], [20]. *F. graminearum* s. str. is the dominant FGSC species associated with head blight in North America [21], whereas *F. asiaticum* appears to be the major species in temperate regions of Asia [3], [6]. Members of *F. graminearum* complex cannot be differentiated by morphology. Zhang *et al.* used *Tri101*, reductase and histone H3 genes on a small set of isolates to identify FGSC species and indicated that most Chinese strains were either *F. graminearum* s. str. or *F. asiaticum* [4]. However, the sequence information from these genes is insufficient to accurately distinguish all the species. Yang *et al.* designed a set of primers to detect 3 species in a single PCR assay and screened a large number of isolates collected from barley in southern China, showing that *F. asiaticum* was the predominant pathogen [3]. However, several isolates could not be characterized in this way. Whether other species of the FGSC or other *Fusarium* species occur in China is currently unknown. Further species identification of FHB pathogens in China is needed to refine the knowledge on the distribution of the 15 species of the FGSC. This information will support plant quarantine programs aimed at preventing the introduction of foreign FHB species across the globe, as was recently reported in Southern USA [22].

The potential presence of hybrids within the *F. graminearum* complex is important to understand fungal evolution. Interspecific crossing *in vitro* was successful in the *F. graminearum* complex [23]. However, there are no reports about the natural occurrence of interspecific hybrids. So far, the only exception reported is a single hybrid between *F. meridionale* and *F. asiaticum* found in Nepal [24]. No hybrids were detected in New Zealand [25] or Japan [6], where two FGSC species co-occur.

Variable number of tandem repeat (VNTR) markers are used regularly in population analysis of *F. graminearum* [1], [2], [26], [27]. Genotyping using VNTRs is a sensitive, fast, and cost-effective method to measure genetic variability of organisms, and is a preferred method for genetic analysis. The VNTR marker data are easy to obtain and allow co-dominant scoring of alleles [28]. VNTR analysis was previously performed on *F. asiaticum* isolates collected from barley from 18 counties in seven provinces in southern China [1]. However, in terms of the economic importance and acreage, wheat is much more important than barley in China. Further analysis of strains collected from wheat distributed over a broad range of sampling sites is needed to understand the population dynamics of FHB pathogens in small grain cereals.

Individual FGSC isolates produce different trichothecenes: NIV and acetylated derivatives (NIV chemotype), DON and primarily 3-acetyldeoxynivalenol (3ADON chemotype), or DON and primarily 15-acetyldeoxynivalenol (15ADON chemotype) [29]. These chemotypes may affect species or population ecology because the corresponding mycotoxins differ in toxicity and bioactivity [30], [31]. It was hypothesized that chemotype polymorphism is trans-specific and has been maintained through multiple speciation events, indicating that chemotype differences may have a significant impact on pathogen fitness [32]. In previous studies, all three trichothecene chemotypes have been found in *F. asiaticum* and only 15ADON was found in *F. graminearum* s. str. in China [4]. Recently, we observed a dramatic increase of 3ADON producers collected from barley along the Yangtze River [1]. We noticed an influx of 3 ADON producers, displacing the old NIV-producing population. However, the driving force behind this sweep is unknown. Whether these 3ADON producers have some selective advantages, and whether the situation for wheat in China shows similar characteristics, is still largely unknown.

In this study, we used multilocus genotyping (MLGT) [21] to determine both the species and the chemotype of FHB pathogens collected from symptomatic wheat from 175 sampling sites in 15 provinces. Based on six genes, 13 FGSC species, five closely related *Fusarium* species and three chemotypes were detected simultaneously with each species and chemotype being identified by probes in two different genes. We also investigated the possibility of natural hybridization between *F. graminearum* s. str. and *F. asiaticum* in China. In addition, using VNTR markers, the population structure of *F. graminearum* s. str. and *F. asiaticum* was characterized from nearly all regions in China with FHB epidemics. Finally, we tested for differences in seven phenotypic characteristics between the four population clusters identified, which may contribute to differences in fitness or aggressiveness.

## Results

### Collection of *Fusarium* isolates

We obtained a total of 469 single spore strains from 175 sampling sites in 15 provinces, two or three isolates in each sampling site (Table 1). These sampling sites can roughly be grouped into four regions: (i) the region along the middle and lower valleys of Yangtze River (ML Yangtze River region), including Hubei, a small area in the south of Henan province, Anhui, Jiangsu and Zhejiang. This area is in the subtropical monsoon region with a warm and humid climate, where the average multiyear temperatures range from 15.2 to 17.7°C with about 1660–2320 h of sunshine per year. Rice-wheat rotation dominates this ML Yangtze River region. (ii) the southwest region that includes the upper reaches of Yangtze River, covering Chongqing, Sichuan, Yunnan and the southern part of Shaanxi province. In this mountain area temperatures and relative humidity are high with a lot of fog and less light. The average annual temperatures and hours of sunshine hours are about 16–18°C and 1100–1300 h respectively. The crop rotation is more diverse in this area and besides rice and wheat, maize is grown in large acreages. (iii) the Huanghuaihai region, including Shandong, Hebei, Shanxi, most areas in Henan and the northern part of Shaanxi province. Both the terrain and the climate in this area are complex: the most outstanding features are dry and hot summers and cold winters. Wheat-maize rotations dominate this region, while rice is grown on a small scale. (iv) Northeast region, including Liaoning and Heilongjiang province. In this area, in the northeast of China, winters are long and cold and wheat is grown on a limited acreage. Hence wheat grown in these provinces is primarily spring wheat, as opposed to the other three regions, where winter wheat dominates. In the Northeast, the cropping system is complex with major differences between different counties, such as soybean-wheat-maize, soybean- sorghum-wheat, and so on [33].

**Table 1:** Distribution of *Fusarium* isolates in 15 provinces in China.

Wheat growing areas	Province	Sampling sites	*F. asiaticum*	*F. graminearum* s. str.	*F. meridionale*	*F. proliferatum*	*F. culmorum*	*F. cerealis*	*F. avenaceum*	*F. equiseti*	Total
**Northeast region**	**Heilongjiang**	1	0	5	0	0	0	0	0	0	5
	**Liaoning**	1	0	10	0	0	0	0	0	5	15
**Huanghuaihai region**	**Qinghai**	1	0	0	0	0	0	4	0	0	4
	**Shandong**	4	5	15	0	0	0	0	0	0	20
	**Shanxi**	1	0	15	0	0	1	0	0	0	16
	**Hebei**	12	0	42	0	0	0	0	0	0	42
	**Henan**	20	3	35	0	0	0	0	0	0	38
	**Shaanxi (North)**	5	0	7	0	0	0	0	0	0	7
**Southwest region**	**Shaanxi (South)**	9	11	10	0	0	0	0	0	0	21
	**Sichuan**	32	54	16	1	0	0	0	1	0	72
	**Chongqing**	2	17	0	0	0	0	0	3	0	20
	**Yunnan**	1	0	0	1	0	0	6	0	1	8
**ML Yangtze River region**	**Hubei**	20	47	3	0	0	0	0	0	0	50
	**Anhui**	16	30	2	0	0	0	0	0	0	32
	**Zhejiang**	8	26	4	0	0	0	0	0	0	30
	**Jiangsu**	42	82	5	0	2	0	0	0	0	89
	**Total**	175	275	169	2	2	1	10	4	6	469

### Species and trichothecene chemotype determination

Four hundred and fifty-seven isolates could be analyzed by the MLGT assay. In total five *Fusarium* species were detected, of which three species belonged to the FGSC: *F. asiaticum* (n = 275), *F. graminearum* s. str. (n = 169) and *F. meridionale* (n = 2) with an additional set of *F. cerealis* (n = 10) and *F. culmorum* (n = 1). Based on the results of *TEF-1α* gene sequence alignment against FUSARIUM-ID databases, the additional 12 strains were identified as *F. equiseti* (n = 6), *F. avenaceum* (n = 4) and *F. proliferatum* (n = 2). The distribution of *Fusarium* isolates is summarized in Table 1. *F. graminearum* s. str. dominates the Northeast and Huanghuaihai regions, with a total of 129 isolates, and only eight strains of *F. asiaticum*. In the Southwest and ML Yangtze River regions, *F. asiaticum* was the predominant species, with a total of 267 isolates. Next to this we identified 40 *F. graminearum* s. str. strains collected in 30 sampling sites in these two regions. The two *F. meridionale* isolates were obtained from Sichuan and Yunnan. A small set of *F. cerealis* (n = 10) was found in Qinghai (n = 4) and Yunnan (n = 6), one *F. culmorum* isolate was sampled from Shanxi. *F. proliferatum* was detected twice at two different sites in Jiangsu. Four *F. avenaceum* strains were isolated from Sichuan (n = 1) and Chongqing (n = 3) respectively. Finally, *F. equiseti* was found five times in Liaoning and once in Yunnan (Table 1).

Results of the trichothecene chemotype determination are shown in Table 2. All *F. graminearum* s. str. strains had the 15ADON chemotype. In *F. asiaticum* isolates we detected all three chemotypes. 3ADON and NIV were the main chemotypes represented by 171 and 97 isolates, respectively. In addition, a small set of seven *F. asiaticum* isolates with the 15ADON chemotype was found, that was collected in Sichuan, Hubei and Zhejiang. NIV and 3ADON producers showed a strong association with their geographical origin. Over 80% of the *F. asiaticum* isolates collected from the Southwest region were NIV producers. In contrast, along the middle and lower valleys of the Yangtze River, the percentage of 3ADON producers was high (83%). Both *F. meridionale* and all *F. cerealis* strains had the NIV chemotype, while the single *F. culmorum* strain, that originated from Shanxi, had the 3ADON chemotype.

**Table 2:** Trichothecene type compositions of *F. graminearum* s. str. and *F. asiaticum* in China.

Wheat growing areas	Province	No. of isolates	*F. asiaticum*			*F. graminearum* s. str.
			NIV^a^	3ADON^a^	15ADON^a^	15ADON^a^
**Northeast region**	Heilongjiang	5	0	0	0	5
	Liaoning	10	0	0	0	10
**Huanghuaihai region**	Hebei	42	0	0	0	42
	Shanxi	15	0	0	0	15
	Shandong	20	0	5	0	15
	Henan	38	2	1	0	35
	Shaanxi (North)	7	0	0	0	7
**Southwest region**	Shaanxi (South)	21	10	1	0	10
	Sichuan	70	44	6	4	16
	Chongqing	17	14	3	0	0
**ML Yangtze River region**	Hubei	50	3	42	2	3
	Anhui	32	4	26	0	2
	Jiangsu	87	11	71	0	5
	Zhejiang	30	9	16	1	4
	Total	444	97	171	7	169

a Trichothecene type was determined by MLGT assay (Ward *et al.* 2008).

### Population analyses

Twelve VNTR markers were used to genotype the *F. graminearum* s. str. and *F. asiaticum* strains (n = 444). In initial simulations in STRUCTURE 2.2, we assumed a genetic cluster number between two and five (K = 2−5). Three clusters were made with the highest increase in probability while further increases in K resulted in additional clusters with lower posterior probabilities. Isolates were assigned to a specific population when probability membership values were higher than 0.8 (*q*≥0.8). Nearly 90% of the isolates could be grouped in three genetically distinct clusters, POP1, POP2 and POP3, respectively (Table 3). A high level of population genetic differentiation (*Fst*, 0.227 to 0.337) combined with a low number of migrants (*Nm*, 0.984 to 1.703) across the three populations was detected (Table 4). The values of genetic differentiation were all statistically significant (*P*<0.001). The population pair formed by the POP1 and POP2 populations showed the highest *Fst* and lowest *N*m (Table 4).

**Table 3:** Isolate compositions of three genetic clusters defined by high probability membership values (q≥0.8).

Wheat growing areas	Province	species^a^	chemotype^a^	Genetic clusters^b^				Total
				POP1	POP2	POP3	Admixed	
**Northeast region**	Heilongjiang	*F. graminearum* s. str.	15ADON	4	0	0	1	5
	Liaoning	*F. graminearum* s. str.	15ADON	9	0	0	1	10
**Huanghuaihai region**	Hebei	*F. graminearum* s. str.	15ADON	42	0	0	0	42
	Shanxi	*F. graminearum* s. str.	15ADON	15	0	0	0	15
	Shandong	*F. graminearum* s. str.	15ADON	15	0	0	0	15
		*F. asiaticum*	3ADON	0	5	0	0	5
	Henan	*F. graminearum* s. str.	15ADON	33	0	0	2	35
		*F. asiaticum*	3ADON	0	1	0	0	1
			NIV	0	0	1	1	2
	Shaanxi (North)	*F. graminearum* s. str.	15ADON	7	0	0	0	7
**Southwest region**	Shaanxi (South)	*F. graminearum* s. str.	15ADON	10	0	0	0	10
		*F. asiaticum*	3ADON	0	1	0	0	1
			NIV	1	1	8	0	10
	Sichuan	*F. graminearum* s. str.	15ADON	14	0	0	2	16
		*F. asiaticum*	15ADON	0	1	2	1	4
			3ADON	2	1	1	2	6
			NIV	1	8	26	9	44
	Chongqing	*F. asiaticum*	3ADON	0	2	0	1	3
			NIV	0	3	6	5	14
**ML Yangtze River region**	Hubei	*F. graminearum* s. str.	15ADON	3	0	0	0	3
		*F. asiaticum*	15ADON	1	0	0	1	2
			3ADON	1	34	4	3	42
			NIV	1	1	1	0	3
	Anhui	*F. graminearum* s. str.	15ADON	1	0	0	1	2
		*F. asiaticum*	3ADON	0	25	0	1	26
			NIV	0	3	1	0	4
	Jiangsu	*F. graminearum* s. str.	15ADON	5	0	0	0	5
		*F. asiaticum*	3ADON	0	61	1	9	71
			NIV	0	4	3	4	11
	Zhejiang	*F. graminearum* s. str.	15ADON	0	3	1	0	4
		*F. asiaticum*	15ADON	0	1	0	0	1
			3ADON	0	11	2	3	16
			NIV	0	3	5	1	9
			Total	165	169	62	48	444

a Species and trichothecene type was determined by MLGT assay (Ward *et al.* 2008).

b Genetic clusters were divided with STRUCTURE 2.2 (Falush *et al.*, 2003; Pritchard *et al.*, 2000).

**Table 4:** Pairwise comparisons of effective number of migrants (*N*m), above diagonal, and genetic differentiation (*F*st), below diagonal, among 3 populations.

Population	POP1	POP2	POP3
**POP1**	…	0.984	1.338
**POP2**	0.337*	…	1.703
**POP3**	0.272*	0.227*	…

*indicates significant at *P*<0.001.

Altogether, 48 isolates (11%) that could not be placed into one of the three populations described above and these were named admixed strains. Of 41 *F. asiaticum* admixed strains, the *q* value assigned to POP1 population was very low (0.029±0.005), and there were no significant differences between *q* values assigned to either POP2 (0.542±0.032) or POP3 (0.428±0.033). However, for seven *F. graminearum* s. str. admixed isolates, high *q* values (0.763±0.008) were observed, when they were assigned to the POP1 population. This indicated little hybridization between the two species. In other to have a closer look at possible hybrids between *F. graminearum* s. str. and *F. asiaticum* we performed a PCR-RFLP analysis.

All *F. graminearum* s. str. and *F. asiaticum* isolates were subjected to PCR-RFLP to detect potential hybrids between the two species. Based on the results of 15 PCR-RFLP markers tested, all *F. graminearum* s. str. isolates showed specific patterns for *F. graminearum* s. str., whereas all *F. asiaticum* isolates exhibited F. *asiaticum* profiles. No hybrid patterns were identified among the 444 strains from China, including the 27 sampling sites where both *F. graminearum* s. str. and *F. asiaticum* were identified in this study.

**Figure 1:**
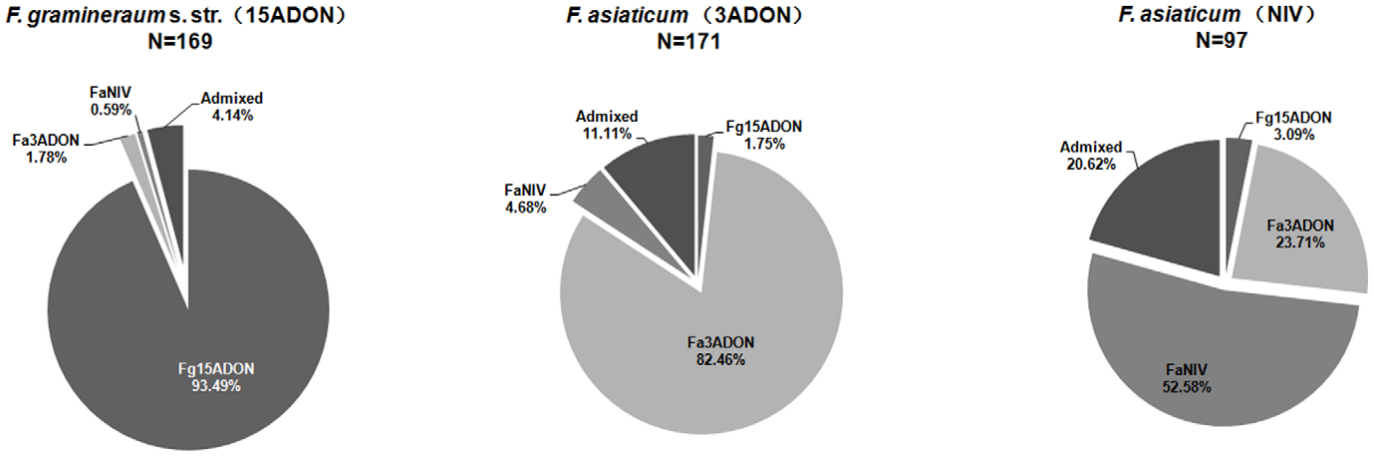
Genetic clusters compositions of *F. graminearum* s. str. with 15ADON chemotype (N = 169), *F. asiaticum* with 3ADON chemotype (N = 171) and with NIV chemotype (N = 97).

The three populations identified by STRUCTURE analysis showed significant correlation with both species and chemotype. Most *F. graminearum* s. str. isolates (93.5%) were assigned to POP1. For *F. asiaticum* strains with the 3ADON chemotype, 82.5% were assigned to the POP2. Unlike the two groups above, of which most could easily be assigned to one specific population, the genotypes of *F. asiaticum* strains with NIV chemotype showed clear differentiation. Only half of the isolates (52.6%) fit into POP3 population, however, up to 23.7% had genotypes consistent with POP2 population which mainly consisted of 3ADON producers. There were also 20.6% admixed genotypes, which was also significantly higher than the ratio of 3ADON producers with admixed genotype (*P*<0.05, Figure 1). We also performed a STRUCTURE analysis on the *F. graminearum* s. str. and *F. asiaticum* isolates individually. For *F. asiaticum*, 90.0% (N = 247) isolates were grouped to two clusters, FaPOP1 (N = 180) and FaPOP2 (N = 67) and the other 28 isolates (10.0%) that could not be placed into one of the two populations were named FaMIX strains. High population genetic differentiation (*Fst* = 0.208) combined with a low number of migrants (*Nm* = 1.904) between them was observed and the variation between populations was high (21%), suggesting they were two distinct clusters. The composition of the two clusters were high consistent with POP2 and POP3, all the strains in POP2 and POP3 were assigned to FgPOP1 and FgPOP2 respectively in this analysis. For *F. graminearum* s. str., 85.8% (N = 145) isolates were grouped to two clusters, FgPOP1 (N = 65) and FgPOP2 (N = 80), the others (N = 24) were FgMIX strains. However, different from *F. asiaticum*, we detected much lower population genetic differentiation (*Fst* = 0.102) and higher number of migrants (*Nm* = 4.390) between them, the variation between populations was only 10.0%, most of the variation (90.0%) was observed among individuals within populations. These results reveal that these two populations are genetically closely related, but infrequent recombination between them has maintained the integrity of the population.

The genetic subdivision within *F. graminearum* s. str. isolates, was further assessed by analysis of molecular variance (AMOVA) on *F. graminearum* s. str. strains from the main regions of origin (Heilongjiang, Liaoning, Hebei, Shandong, Shanxi, Henan, Shaanxi, Sichuan). In the AMOVA analysis, isolates were grouped according to the province from which they were collected, except for the provinces Heilongjiang and Liaoning, which were combined to compensate forthe low number of isolates. This analysis resulted in only limited variation (7.3%) between the seven provinces (*F*st = 0.073), while most of the variation (92.7%) was observed among individuals within a province. Moreover, the linkage disequilibrium (LD) value within the provinces was low (0.022).

*F. asiaticum* dominated the Southwest region and the ML Yangtze River region. To obtain more detailed information on the genetic subdivision and to identify potential migrants among the *F. asiaticum* strains (POP2 and POP3), smaller regions from west to east were compared: Southwest region, Hubei, Anhui, Jiangsu and Zhejiang. Genotypic diversity (*GD*) was high in all five regions, ranging from 0.997 to 1.000 (Table 5). Gene diversity (*H*) ranged from 0.5057 (Anhui) to 0.6220 (Southwest region). Significant linkage disequilibrium (*P*<0.01) was observed in the Southwest (0.075) and in Hubei (0.074), the LD value at the other three regions was very low, ranging from 0.005 (Zhejiang) to 0.013 (Anhui). This indicates that the LD value increased from east to west along the Yangtze River. The percentage of NIV producers in POP2 was low in the Southwest region (17.7%) and Hubei (33.3%), but much higher in Anhui (75.0%), Zhejiang (33.3%) and Jiangsu (36.4%) and showed an opposite trend compared to the LD value (Table 5). The Southwest region also revealed the highest proportion (22.0%) of admixed strains. We performed similar calculations for POP2 and POP3. The gene diversity (*H*) was 0.534 and 0.561 for POP2 and POP3 respectively. Significant linkage disequilibrium (*P*<0.01) was observed in POP3 (0.055), however, the LD value of POP2 was much lower (LD = 0.003, *P* = 0.198).

**Table 5:** Multilocus linkage disequilibrium (LD) and percentage of NIV producers in POP2 genotype of *F. asiaticum* in five regions.

Population	Isolates	*GD* a	*H* b	LD^c^	Percentage of NIV producers in POP2	Percentage of admixed strains
**Southwest**	82	0.999	0.6220	0.075*	17.7%	22.0%
**Hubei**	47	0.998	0.5689	0.074*	33.3%	8.0%
**Anhui**	30	0.997	0.5057	0.013	75.0%	6.3%
**Jiangsu**	82	0.999	0.5182	0.007	36.4%	14.9%
**Zhejiang**	26	1.000	0.5759	0.005	33.3%	13.3%

a Genotypic diversity (*GD*).

b Gene diversity (*H*).

c Measure of multilocus LD.

*indicates significant at *P*<0.01.

### Phenotypic analyses

To get a better understanding of the basis of the genetic subdivision and the dynamics of the pathogens, we tested whether phenotypic differences between the strains could contribute to fitness or aggressiveness. This analysis was performed on four populations: POP1, POP2A (*F. asiaticum* isolates with the 3ADON chemotype, the predominant chemotype of POP2), POP2B (*F. asiaticum* isolates with the NIV chemotype that genetically fit into the POP2 population based on the VNTR markers) and POP3. Although isolates were divided into two populations in the individual VNTR analysis of *F. graminearum* s. str., the low value of population genetic differentiation, variation between populations, linkage disequilibrium and high number of migrants indicated both chemotype and geographical distribution showed no correlations with the clusters. Also, AMOVA revealed no significant variation among *F. graminearum* s. str. collection sites. So in the phenotypic analyses, POP1 was considered as a single population. From each population twenty strains were selected randomly. The incidence of infected spikelets (IIS) was significantly higher (*P*<0.05) for POP2A and POP1 compared to POP3 and POP2B (Table 6). Similarly, the biomass of the infecting pathogens as determined by the amount of DNA was significantly higher (*P*<0.05) in POP2A (56.5±6.0) and POP1 (57.4±3.1) compared to POP3 (29.7±2.7) and POP2B populations (32.5±1.5) (Table 6). These results illustrated that the POP1 and POP2A populations were more aggressive to wheat than the other two populations.

**Table 6:** Population mean and standard error estimates for incidence of infected spikelets^a^, biomass, growth, conidia, resistance to MBC and trichothecene accumulation (ppm).

Phenotypic tests	POP1	POP2A	POP2B	POP3
**Yangmai 158 7 dpi (IIS)**	8.6±0.4	9.5±0.7	6.8±0.1	6.9±0.1
**Annong 8455 7 dpi (IIS)** b	35.5±1.6a	33.9±2.1a	14.1±1.4b	14.7±1.9b
**Yangmai 158 14 dpi (IIS)** b	23.2±1.4a	22.9±2.0a	9.6±0.6b	9.7±0.7b
**Annong 8455 14 dpi (IIS)** b	58.5±1.2a	55.9±1.7a	34.3±2.7b	35.2±1.9b
**Yangmai 158 21 dpi (IIS)** b	55.0±2.0a	51.3±2.4a	32.9±3.7b	34.6±2.7b
**Annong 8455 21 dpi (IIS)** b	76.2±1.7	79.8±1.3	66.9±1.1	68.3±2.2
**Biomass (ng/mg tissue)**	56.5±6.01a	57.43±3.11a	29.73±2.69b	32.52±1.5b
**Growth rate (mm/d)**	8.7±0.26b	9.25±0.48a	7.71±0.37c	7.99±0.32c
**Conidia produced (thousand)**	365.95±36.71a	211.1±25.42b	124.53±14.52c	198.13±13.84b
**Conidial length (µm)**	50.13±1.35a	43.12±1.22b	37.74±0.77c	42.26±0.83b
**Resistance to MBC (IC50)**	0.561±0.021b	0.623±0.021a	0.488±0.014c	0.582±0.016ab
**DON** b	406.1±43.8a	350.9±42.9a	ND	ND
**15ADON** b	32.9±6.4b	82.6±11.8a	ND	ND
**3ADON** b	9.8±2.9b	244.2±29.3a	ND	ND
**NIV** b	ND^c^	ND	79.8±7.9a	66.7±7.5a
**Total Trichothecene accumulation**	443.3±52.4b	683.2±76.5a	79.8±7.9c	66.7±7.5c

a IIS is the incidence of infected spikelets. For statistical analysis, IIS data were arcsine transformed and the values presented are back-transformed means.

b Values within a row followed by different letters are significantly different at *P*<0.05.

c ND indicated not detected.

In trichothecene accumulation tests, isolates that belong to the POP2A population produced significantly more mycotoxin than the other three populations (*P*<0.05), with a total amount of 683.2±76.5 ppm. The total trichothecene accumulation of isolates that belonged to the POP1 population (443.3±52.4 ppm) was higher (*P*<0.05) than the two NIV producing populations, while there was no significant difference between POP3 (79.8±7.9 ppm) and POP2B (66.7±7.5 ppm). We also found no difference in the DON production between POP2A producing population (406.1±43.8 ppm) and the POP1 producing population (350.9±42.9 ppm). However the POP2A producers produced more 3ADON than the POP1 producers produced 15ADON (Table 6).

Isolates that belong to the POP2A population revealed significant greater radial growth rates (9.25±0.48, *P*<0.05) than the other populations. No significant difference in radial growth rates was observed between POP3 (7.71±0.37) and POP2B (7.99±0.32), both of which were significantly lower than POP1 (8.7±0.26, *P*<0.05) (Table 6). The highest numbers of conidia (365.95±36.71, *P*<0.05) and the largest conidia (length: 50.13±1.35, *P*<0.05) were produced by isolates from the POP1 population. There were no significant differences in the amount of conidia nor in their length between the POP2A population (211.1±25.42 and 43.12±1.22) and the POP2B population (198.13±13.84 and 42.26±0.83), but both populations produced significantly (*P*<0.05) higher numbers of conidia, that were also larger as compared to the POP3 population (124.53±14.52 and 37.74±0.77) (Table 6). In the sensitivity test to benzimidazole, six resistant strains were identified (1.4 and 5 µg/ml) all of them were collected from Jiangsu. We tested the effective concentration providing a 50% reduction in radial growth (IC_50_) of 20 sensitive isolates in each population. The POP2A (0.623±0.021) and POP2B (0.582±0.016) populations showed a similar IC_50_ value, and both the IC_50_ of them were significantly (*P*<0.05) higher than POP3 (0.488±0.014). POP1 isolates revealed mediate IC_50_ (0.561±0.021), lower than POP2A, but not significantly different from POP2B population. Comparison of phenotypic differences between four population pairs is summarized (Table 7). The POP2A population showed significant differences to the POP3 population in all seven phenotypic tests. It is interesting that although POP2A and POP2B shared similar genotypes based on VNTR markers, POP2B isolates were comparable in only three phenotypic traits (numbers of conidia produced, conidial length and the resistance to MBC) with the POP2A population, while ISS, biomass, growth rate and mycotoxin production were all significantly higher in the POP2A population and these four traits of POP2B were not significantly different from POP3 population which were also mainly consisted of NIV producers. Compared with the POP2A population isolates of the POP1 population produced more and larger conidia, but less trichothecenes while they also showed slower radial growth.

**Table 7:** Pairwise comparison of the four populations in phenotypic differences.

Population pairs	POP1POP2A	POP2APOP3	POP2APOP2B	POP2BPOP3
IIS	n^a^	+^b^	+	n
Biomass	n	+	+	n
Growth rate	−c	+	+	n
Trichothecene accumulation	n	+	+	n
Resistance to MBC	−	+	n	+
Conidia produced	+	+	n	+
Conidial length	+	+	n	+

a “n” indicated no significant differences found between the two populations.

b “+” indicated the population above significantly higher than the population below at *P*<0.05.

c “−” indicated the population above significantly lower than the population below at *P*<0.05.

## Discussion

According to genealogical concordance with 13 genes totaling 16.3 kb, 15 species were identified within *F. graminearum* complex, most of which cannot be distinguished morphologically [20]. Currently the MLGT assay [21] is the most accurate exact high-throughput method for species recognition. Based on this robust method, we identified eight *Fusarium* different species among 469 isolates originating from 175 wheat sampling sites in 15 provinces in China, covering almost all wheat growing areas suffering from FHB in China. *F. asiaticum* and *F. graminearum* s. str. dominated the southern and northern parts of China, respectively, which is consistent with results from previous surveys [3], [4], [34]. Other species were found in lower proportions. The Southwest region showed the highest species diversity, with six species being detected, while in the ML Yangtze River region beside *F. asiaticum* and *F. graminearum* s. str. only *F. proliferatum* was identified. Likewise, *F. equiseti* was identified in the Northeast region, which corroborates previously published work [35]. We also identified several *F. cerealis* isolates, a species that was rarely detected since the first report of three isolates in China in 1991 [29]. A high proportion of *F. cerealis* isolates was found in Yunnan (n = 6) and Qinghai (n = 4), which contrasts to previous studies in which only *F. asiaticum* isolates were found [2]. Due to the limited number of sampling sites in both provinces, further surveys are needed to obtain larger numbers of isolates in these two provinces to get a better understanding of the importance of *F. cerealis*.

**Figure 2:**
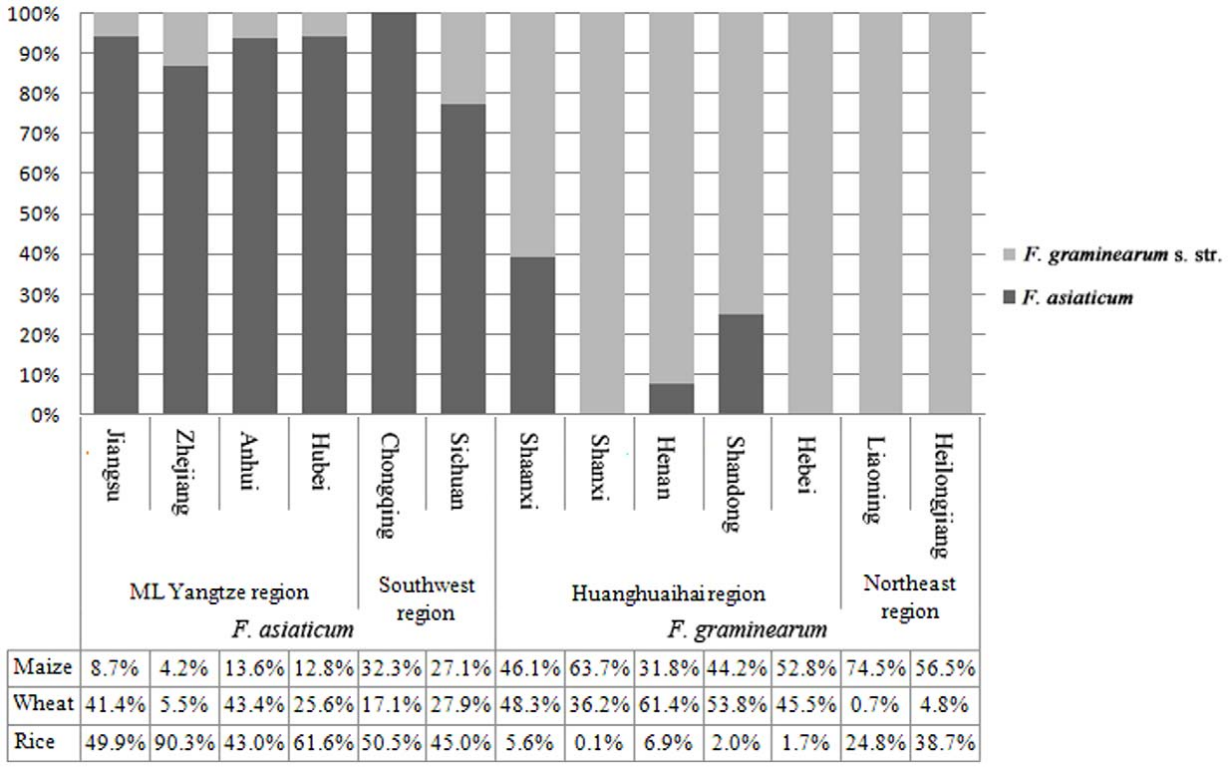
The proportion of wheat, rice and maize planting area and the distribution of *F. graminearum* s. str. and *F. asiaticum* in 13 provinces of four regions.

We found an uneven distribution of *F. graminearum* s. str. and *F. asiaticum* (Figure 2). Qu *et al.* reported that temperature affected the distribution of the two species. *F. graminearum* s. str. is found in cooler regions where the annual average temperature was 15°C or lower, while the vast majority of *F. asiaticum* isolates were collected from warmer regions, where the annual average temperature is above 15°C [14]. In this study, we identified many *F. graminearum* s. str. isolates in the Southwest region, 22.2% in Sichuan and nearly 50% in south Shaanxi, significantly more than in ML Yangtze River region (7.0%). A similar result was also reported for isolates collected from barley [3]. However, no significant temperature differences were observed between these two regions, the average temperature of Southwest region was even a little higher [33]. This indicates that temperature may not be the critical factor in the distribution of the *Fusarium* species and that other, yet unknown factors affected their distribution. Maize residues and rice straw seem critical for overwintering of the pathogen. In Korea, a predominance of lineage 6 of *F. graminearum* (equivalent to *F. asiaticum*) over lineage 7 of *F. graminearum* (equivalent to *F. graminearum* s. str.) was hypothesized to be due to a superior fitness on rice [36] and in southern USA, the occurrence of *F. asiaticum* neatly overlaps with rice-growing areas in Louisiana [22].

We collected the acreage data of rice, wheat and maize from 2005–2009 (data from National Bureau of Statistics of China), and found a strong association between the occurrence of *F. asiaticum* and/or *F. graminearum* and the predominant crops (Figure 2). In the Huanghuaihai region, where maize-wheat rotation is the main cropping system and the acreage of rice is less than 7%, *F. asiaticum* strains were rare. In northern China where rice is not rotated with wheat in the same field, we also did not detect *F. asiaticum*. In the ML Yangtze region, the percentage of maize acreage ranged from 4.2% in Zhejiang to 13.6% in Anhui, and *F. graminearum* s. str. was found in a few sampling sites. In Sichuan province, where rice (45.0%) and maize (27.1%) acreages were both high, a higher proportion of *F. graminearum* s. str. isolates was obtained as compared to other provinces in southern China.

The distribution of different chemotype strains was in agreement with previous surveys in barley [1], [3] and wheat [37]. No *F. graminearum* s. str. strains with 3ADON type were found among any of the 175 sampling sites, including Handan where two strains of the 7C1 population (equivalent to *F. graminearum* s. str.) with 3ADON type were found on maize [37]. Ward *et al.* reported that in North America, *F. graminearum* s. str. strains with 3ADON and 15ADON chemotype belong to different populations and that isolates from the 3ADON population were more aggressive and seem to produce larger amounts of toxin compared to isolates from the 15ADON population. Therefore, monitoring the spatial and temporal dynamics of this high-risk population in China is very important. Once spread, it may cause serious losses and aggravate health hazards in northern China. NIV and 3ADON producers of *F. asiaticum* were predominant in Southwest region and ML Yangtze region, respectively. *F. asiaticum* with the NIV chemotype was recently isolated in America in regions were rice was grown [22]. The intraspecific variation of the US *F. asiaticum* population was found to be low, which would indicate a recent introduction into the United States. Our data also indicate that the growth of rice may be a key factor for the presence of *F. asiaticum*. However, in China we found no association between the NIV chemotype and the cultivation of rice, which was observed for the southern part of the USA by Gale et al. [22]. No *F. graminearum* isolates producing NIV were found and also in regions where rice is the dominant crop, we mostly found *F. asiaticum* with the 3ADON chemotype. In China, *F. asiaticum* isolates that produce NIV were mainly found in the Southwest region and appear to be the remainder of an old population that is currently replaced [1], [2]. Different continents may show different trends and, as shown in this study, the chemotype may not be the only factor driving the replacement.

We found that *F. asiaticum* and *F. graminearum* s. str. co-occurred in 24 sampling sites. Hybrids between *F. graminearum* s. str. and *F. asiaticum* were previously generated by *in vitro* crosses [23], indicating that gene flow may occur between these species. Indeed, a natural hybrid between *F. meridionale* and *F. asiaticum* was found in Nepal [24]. However, based on the strong genealogical concordance across multiple loci, O'Donnell *et al.* [24] concluded that hybridization among different species of the *F. graminearum* complex was not frequent enough to counteract differentiation by genetic drift. Similar to surveys in New Zealand [25] and Japan [6], no hybrid was detected with PCR-RFLP makers in China including 27 areas, where the two species co-occurred. Our population analyses further strengthen the lack of hybrids. In the 41 *F. asiaticum* admixed isolates, the *q* value mainly contributed to POP2 and POP3 populations, while in seven *F. graminearum* s. str. admixed strains, all *q* value assigned to POP1 was high (*q*>0.7). This suggests that hybridization is common between POP2 and POP3 populations which were mainly consisted of *F. asiaticum* isolates with 3ADON and NIV type respectively, but not between *F. asiaticum* (POP2 and POP3) and *F. graminearum* s. str. (POP1). In addition, the trichothecene chemotype composition was significantly different between *F. graminearum* s. str. and *F. asiaticum* (Table 2). All *F. graminearum* s. str. isolates were 15ADON chemotype, while 97.5% *F. asiaticum* were NIV- or 3ADON-producers. All of this strongly indicates that some unknown barriers limited the gene flow between these species in nature.

**Figure 3:**
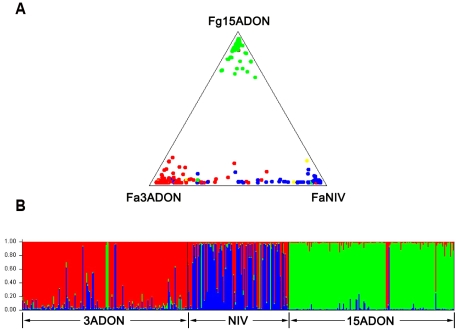
Visual representation of isolate distribution (A) and admixture estimates (B) based on VNTR data. A: green = *F. graminearum* s. str. with 15ADON type, red = *F. asiaticum* with 3ADON type, blue = *F. asiaticum* with NIV type, yellow = *F. asiaticum* with 15ADON type; B. Visual representation of admixture estimates based on VNTR data for 275 *F. asiaticum* and 169 *F. graminearum* s. str. isolates collected from three Chinese populations (3ADON, NIV and 15ADON). Each population is represented by a unique color (3ADON = red, NIV = blue and 15ADON = green). Individual isolates are represented by a distinct vertical line colored to represent the estimated proportion of the isolates genome derived from each population. The horizontal axis consists of a single vertical bar for each of 444 isolates. Isolates were assigned to a specific population when membership fraction ≥0.8.

Recently, a chemotype shift with a new population of *F. graminearum* complex was observed on wheat in North America [21], [38], [39] and on barley in China [1]. We performed VNTR analyses to detect the genetic structure and the potential migration of members of the *F. graminearum* species complex on wheat in China. Allele data were initially analyzed with a Bayesian model using the program STRUCTURE 2.2 [26], [27]. The model is based on maximizing linkage equilibrium within clusters and disequilibrium between clusters and therefore seeks to recreate populations that recombine. For all *F. graminearum* s. str. and *F. asiaticum* isolates, three clusters were identified from the data and most isolates were assigned to a specific population with high probability values (*q*≥0.8). High levels of population genetic differentiation (*F*st) and low levels of effective numbers of migrants (*N*m) were observed between these clusters, indicating they represented distinct populations with limited genetic exchange among them. *F. graminearum* s. str. isolates were highly concentrated in the POP1 population. Based on the absence of hybrids and on the VNTR analysis, the POP1 population was shown to be independent from the two *F. asiaticum* populations (Figure 3A). In contrast, for *F. graminearum* s. str. we found a low genetic differentiation among isolates collected from different geographic origin (7.34%, *F*st = 0.073) combined with a low multilocus linkage disequilibrium (0.022). In the individual VNTR analysis for *F. graminearum* s. str., isolates were divided into two clusters. However, we detected a low value for population genetic differentiation, variation between populations, linkage disequilibrium and high number of migrants. Also the clusters did not correlate with neither chemotype nor geographical distribution. For the purposes of this study, we therefore regarded the *F. graminearum* s. str. population as a single population.

Karugia *et al.* [27] clustered 478 *F. asiaticum* isolates from China and Japan into 3 populations, each showing significant correlation with chemotype. High levels of pairwise *F*st and low levels of *N*m observed among these populations indicated limited gene flow between them [27]. The current study showed similar results. In the analysis of all *F. graminearum* s. str. and *F. asiaticum* isolates, we divided *F. asiaticum* isolates into two populations with NIV (POP3) and 3ADON (POP2) chemotype using the same method and detected significant *F*st and low *N*m between them. The individual STRUCTURE analysis on *F. asiaticum* showed a similar result. Like Karugia's study, some strains with 3ADON or NIV chemotype were also classified into POP3 and POP2 populations, respectively. Also the two *F. asiaticum* populations showed low *F*st and high *N*m and most admixed isolates were NIV or 3ADON producers, suggesting outcrossing has occurred between them (Figure 3A). However, the outcrossing was unequal, we found a trend that 3ADON and the alleles that are prevalent in the 3ADON producers increase in the population. There is a selective sweep of the 3ADON producers and the genes they carry, which is illustrated by the biased gene flow that was observed from 3ADON to NIV producers (Figure 3B). This biased gene flow is likely due to recent migration of 3ADON isolates by two mechansims. (i) migration of asexual spores or ascospores after selfing. (ii) migration by outcrossing in which case the genes and alleles will mix with the genes and alleles of local isolates. Successful genotypes show enhanced proliferation both by selfing which will generate more spores and by outcrossing, as they can be father and mother. Unsuccessful genotypes may not have the resources required to support the formation of progeny as a mother. Therefore, these successful genotypes and the prevalent alleles they carry will increase in the population. This is in agreement with the finding on barley in China [1], but the trend on wheat was even stronger. The high *GD*, *H* and low LD value in all five areas in Southwest and ML Yangtze River regions suggested frequent outcrossing occurred among them and the migration of isolates was possible. The percentage of NIV producers assigned to POP2 decreased from East (Jiangsu) to West (Southwest region), implying the direction of migration was from ML Yangtze region to Southwest region. Also, an increase of the LD value from East to West supported this hypothesis, where the significant higher (*P*<0.01) LD estimates in Southwest and Hubei regions indicate that there has been insufficient time for random mating to break down the LD created by recent migrants. The linkage disequilibrium of the two *F. asiaticum* populations showed the same result, we detected very low and not significant (*P* = 0.198) LD value of POP2 and significant (*P*<0.01) LD of POP3 which was mainly consisted of NIV producers from Southwest region, suggesting the insufficient recombination of isolates in POP3. The high *GD*, *H*, LD and proportion of admixed isolates all suggest that *F. asiaticum* isolates with the 3ADON chemotype may have migrated recently to Sichuan.

Ward *et al.* reported that *F. graminearum* isolates from 3ADON populations produced significantly more trichothecene and had significantly higher fecundity and growth rates than isolates from the 15ADON population in Canada [21]. We performed seven phenotypic tests on the three populations and another population of NIV producers assigned to POP2. Isolates from the POP2A population (3ADON producers) performed significantly better than those from the POP3 population (NIV producers) in all tests. They are more aggressive, more toxigenic, produced more and larger conidia, and had higher growth rates and were resistant to higher concentrations of benzimidazole. Recently, Li *et al.* reported that 3ADON producers are more virulent than NIV producers in the Yangtze basin [40]. Also, Gale *et al.* found that DON producers synthesized approximately four times more toxin and spread significantly faster in the wheat head than NIV producers [22]. In order to know whether there were phenotypic differences between NIV and 3ADON producers in one population, we compared POP2A and POP2B in the seven tests. Although assigned to one population, NIV producers (POP2B) showed significant lower values in four phenotypic tests (ISS, biomass, growth rate and mycotoxin production) than 3ADON producers (POP2A) and these four tests were not significantly different from POP3 population which also NIV producers dominated. The other three tests (conidia produced, conidial length and resistance to MBC) of POP2B were comparable with POP2A. We infer that insufficient time has passed to complete the change in these populations and the members of the admixed population, that would be the result of recombination outcrossing between members of the two populations, therefore show an intermediate phenotype.

In this study we demonstrate that a highly toxigenic and aggressive *F. asiaticum* population, producing 3ADON, is spreading from East to West. These results have important implications for the control of scab and the reduction of trichothecene accumulation on wheat in China. Our findings suggest that adaptations in FHB populations emphasize the need to consider pathogen variation in addition to host variation in FHB management.

## Materials and Methods

### Fungal isolates

**Figure 4:**
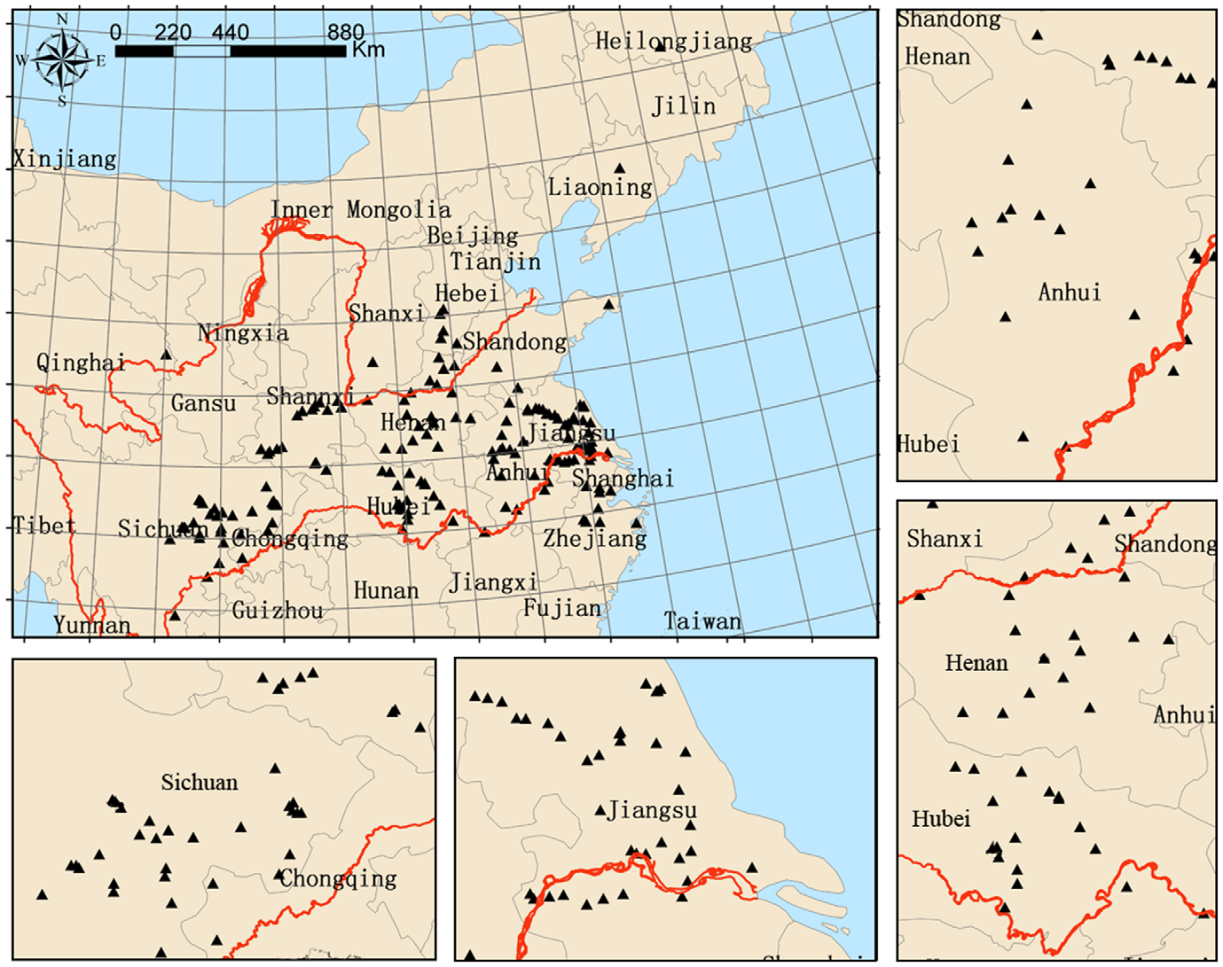
Map of China indicating the 175 sampling sites in 15 provinces.

Diseased wheat spikes with mature kernels were collected from 175 sampling sites in 15 provinces in 2008 (Figure 4). Four or five spikes with FHB symptoms were collected individually at different positions in each field. Kernels of collected wheat spikes were surface sterilized in 70% ethanol for 30 s, and immediately immersed in 2% sodium hypochlorite for 90 s, after which the kernels were extensively rinsed with sterile distilled water and placed on potato dextrose agar (PDA) plates. After 3 days of incubation at 26°C, newly grown-out mycelium was transferred into flasks containing 30 ml of mung bean broth cultures (3% mung bean extract). Flasks were shaken at 180 rpm for 3 days at 26°C. Spore suspensions were diluted to 100-fold and plated on 1% water agar. The plates were incubated for 12 to 24 h and single spores were identified and transferred to PDA plates. Single spore cultures were stored in 15% dimethyl sulfoxide (DMSO) at −80°C. (Strain information is provided in Table S1)

### Genomic DNA extraction

A small mycelial plug was transferred to 6 cm petri plates containing potato dextrose agar (PDA) and incubated at 26°C for 5 to 7 days. Mycelium was harvested with scalpel blades and dried at 50°C overnight. The mycelium was lyophilized with liquid nitrogen and ground by vigorous shaking of the blocks in a MiniBeadbeater-96 (Biospec, USA). Total genomic DNA was extracted using the E.Z 96™ Fungal DNA Kit (Omega Biotek) according to the manufacturer's instructions. DNA concentration was determined by NanoVue Plus (GE, USA). Finally, DNA samples were diluted to 5 ng/µl for each sample in 96-well microtiter plates, which were stored at −20°C.

### Species and trichothecene chemotype determination

Multilocus genotyping (MLGT) [18], [19], [20], [21] was done to determine species and trichothecene chemotype of all strains. To this end, we used partial gene sequences of *TEF-1α*, *Red*, *Mat*, *tri101*, *tri3* and *tri12*. Partial translation elongation factor (*TEF-1α*, ∼700 bp) gene sequences were generated of isolates that could not be identified by the MLGT assay. These strains were identified by sequence comparison in the FUSARIUM-ID database (http://isolate.fusariumdb.org/) [41] and/or in GenBank.

### Detection of hybrids between *F. graminearum* s. str. and *F. asiaticum*

Fifteen PCR-RFLP markers developed by Suga *et al.* were used to detect hybrids between *F. graminearum* s. str. and *F. asiaticum* [6]. PCR was performed at 94°C for 2 min, 30 cycles of 94°C for 1 min, 56°C for 1 min, and 72°C for 1 min. After amplification, *Hin*fI (Takara, Japan) was used for digestion of the amplicons. The mixtures were separated on 1% agarose gels.

### VNTR analyses

Twelve VNTR markers were used for isolate genotyping. Ten VNTR markers were developed by Suga *et al.* [28]. The other two markers, HK1003 and Fuss20 were developed by Gale *et al.* [42] and Vogelgsang *et al.* [43] respectively. All forward primers were labeled with either fluorescent compounds 6-FAM or TAMRA. The PCR mixture was 20 µl containing 10 mM Tris-HCl (pH 8.3), 50 mM KCl, 1.5 mM MgCl_2_, 200 µM dNTP, 1 µM of each primer, 0.5 unit of Taq DNA polymerase (Takara, Japan), and 10 ng of genomic DNA. PCR was carried out in a Mastercycler gradient (Eppendorf, Germany), using the following cycling parameters: 94°C for 1 min; then, 30 cycles of 95°C for 30 s, 58°C for 30 s, and 72°C for 30 s; and a final extension at 72°C for 5 min. Reaction products were scored relative to a GS500 ROX (Applied Biosystems) internal size standard using an ABI 3730 Genetic Analyzer. The data were processed using the genotyping software Gene Mapper v3.5.

VNTR allele data were analyzed by the program STRUCTURE 2.2 [44], [45], which implements a Bayesian model-based clustering method for inferring population structure using genotype data. It assigns multilocus genotypes probabilistically to user-defined K clusters. The genetic clusters are identified by a set of allele frequencies at each locus and by linkage equilibrium within clusters and disequilibrium between clusters. First, we used all *F. asiaticum* and *F. graminearum* s. str. isolates (N = 444) for the analysis. In exploratory runs, we allowed for values of K from 1 to 5, with 10,000 iterations using a Markov chain Monte Carlo (MCMC) method, after a burn-in period of 10,000. The admixture model was selected, because with this method, individuals that have inherited some fraction of the genome from ancestors in different k populations are identified. The parameter of individual admixture, α, was allowed to be different for individual clusters. During exploratory runs, a variety of values for α were used. The final value of α was set to 0.06, because it placed most individuals into specific clusters while still efficiently excluding individuals with admixed genotypes. Allele frequencies were assumed to be uncorrelated between populations. Otherwise, default settings were used. STRUCTURE analysis was also done individually for *F. graminearum* s. str. and for *F. asiaticum*, the parameters were the same as above.

The analysis of molecular variance (AMOVA) was calculated by Arlequin 3.1 [46]. Pairwise *F*st distance estimates were based on the number of different alleles; the statistical significance of pairwise *F*st estimates was assessed using a permutation test with 1,000 permutations. Gene diversity (*H*) was calculated in the program POPGEN version 1.32 (available at http://www.ualberta.ca/~fyeh/). Genotype diversity (*GD*) and multilocus linkage disequilibrium (LD) were estimated by Multi-locus 1.3 [47]. *GD* reveals the probability that two individual strains taken at random have different genotypes. The LD measures the nonrandom association of alleles at different gene loci in a population. The test of significance was determined by using 1,000 randomizations in all populations.

### Pathogenicity analyses

A medium resistant winter wheat cultivar Yangmai 158 and a susceptible cultivar Annong 8455 was selected for the pathogenicity tests. Each cultivar was planted in three different blocks (2 m×4 m) according to normal agronomic practices at the Langfang Farm (Experiment Farm of Institute of Plant Protection) in 2010. We selected twenty strains from each population randomly for pathogenicity analyses. At anthesis, five heads of each cultivar in each block were inoculated by injecting 20 µl conidia suspension (10^6^ conidia/ml) of one isolate into a central floret of a spike (injection inoculation). A total of 30 heads (15 in each wheat cultivar) was infected by a single strain. Pathogenicity was assessed as the incidence of infected spikelets and the biomass of pathogens infected in field grain. The incidence of infected spikelets was determined visually by counting the number of infected spikelets per head at 7, 14 and 21 days after inoculation respectively and was expressed as percentage of total spikelets. The diseased ears of Yangmai158 were harvested to quantify the FHB pathogens using real-time quantitative PCR as described by Waalwijk *et al.* [48]. Langfang Farm is the experiment farm of Institute of Plant Protection and not privately-owned or protected. This field study did not involve endangered or protected species. So no specific permits were required for the described field studies.

### Trichothecene production analysis

The strains tested were cultured on sterile rice kernels. Twenty-five grams of rice grain was soaked in 15 ml of water prior to autoclaving in 150 ml Erlenmeyer flasks. Each flask was inoculated with five pieces of culture (6 mm) grown on PDA. Each flask was shaken once a day for the first 3 days and several times during the remaining days, and was kept up to 2 weeks after inoculation at 25 to 27°C. Each isolate had three replicates. Samples were ground with an A11 mills (IKA, German) and then sonicated in 80 ml of acetonitrile/water (80∶20, vol/vol). After passage over a Bond Elut Mycotoxin column (Varian, USA), trichothecenes were quantified by Agilent Technologies 1200 Series HPLC system with an Extend-C18 column (250 mm×4.6 mm, 5 µm). The binary mobile phase consisted of solvent A (acetonitrile) and solvent B (water) and the gradient program was 15% A at 0 min, 15% A at 8 min, 25% A at 10 min, 25% A at 20 min, and 15% A at 22 min. There was a 2 min post-run under starting conditions for re-conditioning. The flow rate was 0.8 ml/min and the detector was set at 218 nm. Standards for DON, 3ADON, 15ADON, and NIV were obtained from Sigma Aldrich (Chicago, IL).

### Growth and conidiation

A mycelia plug (6 mm in diameter) of one strain from the growing edge of a 5-day old colony was transferred onto a potato dextrose agar plates and incubated at 28°C in complete darkness. Radial growth was measured at four different points along the fastest growing portions of the colony at 24 and 72 h. Each isolate had three replicates. In conidiation experiments, a 6 mm mycelia plug from the growing edge of a 5-day old colony was added into 15 ml tubes containing 2 ml of mung bean broth cultures (3% mung bean extract). The tubes were placed in a shaker at about 30° tilt angle and shaken at 200 rpm for 10 days at 28°C. Conidia were counted using a DM4000B microscope (Leica Microsystems GmbH Wetzlar, Germany). Conidial lengths (in micrometers) were measured for t30 conidia from each isolate using the measurement tool of the DM4000B microscope.

### Resistance to benzimidazole

Sensitivity of strains to benzimidazole was performed as described as Chen *et al*. [49]. To select sensitive isolates, all strains were tested at methyl benzimidazole carbamate (MBC) concentrations of 1.4 and 5 µg/ml. The effective concentration giving 50% inhibition (IC_50_) of a set of sensitive strains was tested by PDA amended with MBC at 0, 0.2, 0.4, 0.6, 0.8, 1.0, and 1.2 µg/ml. Each treatment had four replicates.

All data were processed as an analysis of variance. A least significant difference test (*P* = 0.05 or 0.01) was used to determine whether the populations showed significant differences in pathogenicity, biomass, trichothecene production, resistance to benzimidazole, radial growth or spore production. All data analysis was carried out with the statistical software SAS v8 (SAS Institute Inc., Cary, NC, U.S.A.).

## Supporting Information

Table S1 Strains information in this study.
(XLS)
